# Magnetic Cell Centrifuge Platform Performance Study with Different Microsieve Pore Geometries

**DOI:** 10.3390/s20010048

**Published:** 2019-12-20

**Authors:** Xinyu Wu, Zhongyang Bai, Lin Wang, Guangchao Cui, Mengzheng Yang, Qing Yang, Bo Ma, Qinglin Song, Dewen Tian, Frederik Ceyssens, Robert Puers, Michael Kraft, Weisheng Zhao, Lianggong Wen

**Affiliations:** 1 School of Microelectronics, Beihang University, Beijing 100191, China; 18363992458@163.com (X.W.);; 2 Beihang-Goertek Joint Microelectronics Institute, Qingdao Research Institute, Beihang University, Qingdao 266104, China; 3 Qingdao Institute of Bioenergy and Bioprocess Technology, Chinese Academy of Sciences, Qingdao 266101, China; 4 Qingdao Goertek Microelectronics Research Institute Co., Ltd., Qingdao 266104, China; 5 ESAT-MICAS, KU Leuven, Kasteelpark Arenberg 10, B-3001 Leuven, Belgium

**Keywords:** circulating tumor cells (CTCs), microsieves, magnetic separation, rare cells enrichment, point-of-care

## Abstract

The detection and analysis of circulating tumor cells (CTCs) plays a crucial role in clinical practice. However, the heterogeneity and rarity of CTCs make their capture and separation from peripheral blood very difficult while maintaining their structural integrity and viability. We previously demonstrated the effectiveness of the Magnetic Cell Centrifuge Platform (MCCP), which combined the magnetic-labeling cell separation mechanism with the size-based method. In this paper, a comparison of the effectiveness of different microsieve pore geometries toward MCCP is demonstrated to improve the yield of the target cell capture. Firstly, models of a trapped cell with rectangular and circular pore geometries are presented to compare the contact force using finite element numerical simulations. The device performance is then evaluated with both constant pressure and constant flow rate experimental conditions. In addition, the efficient isolation of magnetically labeled Hela cells with red fluorescent proteins (target cells) from Hela cells with green fluorescent protein (background cells) is validated. The experimental results show that the circular sieves yield 97% purity of the target cells from the sample with a throughput of up to 2 μL/s and 66-fold sample enrichment. This finding will pave the way for the design of a higher efficient MCCP systems.

## 1. Introduction

The cause of cancer-related deaths is mostly due to the metastasis of tumor cells. Malignant cells called circulating tumor cells (CTCs) shed from the primary neoplasm to distant organs via the bloodstream, which forms a secondary tumor [1,2,3,4]. It is also believed that CTCs play a critical role as a real-time “liquid biopsy” in prognosis, detection, characterization, and monitoring of many types of cancers [5,6,7,8,9]. The number of CTCs correlates with progression-free survival and overall survival in patients [9,10,11,12,13]. Furthermore, CTC enumeration can be used to estimate the effectiveness of clinical treatment [6,11,13]. Due to the heterogeneity of individual CTC [14,15], genomic detection and molecular analysis of CTCs are performed to develop targeted anticancer therapies for individual patients [12,16,17]. In addition, CTC profiling also provides the opportunity to research dissemination, drug resistance, and disease recurrence [18,19]. However, the number of CTCs in the metastatic patients’ peripheral blood is extremely rare, with only one per 10^9^ blood cells [20,21]. Therefore, to realize an in-vitro, high throughput CTC enrichment device has become an urgent demand for clinical applications.

To date, many research institutions and companies have successfully demonstrated and developed a variety of mechanisms for the separation and detection of CTCs. Label-free separations distinguish CTCs and normal blood cells depending on physical properties such as size [22,23], density [24], deformability [25,26], and dielectric properties [27,28]. Due to their simplicity, cost-effectiveness, high throughput, and that they are damage-free to biological information for further studies, they have become common methods for enriching tumor cells in the laboratory. However, there is no obvious difference in the physical properties of CTCs and leukocytes, coupled with the heterogeneity of CTCs, which would cause extremely low recovery and possibilities of contamination. Separation methods based on biological characteristics, relying on the expression of special proteins on the cell surface, are mainly divided into positive enrichment of the commonly used epithelial cell adhesion molecule (EpCAM) [21,29,30] and negative selection via frequently used CD45 [31,32]. The combination of antibody-modified magnetic nanoparticles (MNPs) [29,33] with size-based microsieve cell enrichment provides new affinity-based separation methods with better selectivity and higher purity.

In our previous study of the Magnetic Cell Centrifugal Platform (MCCP), we successfully demonstrated the enrichment of the target cells based on a combination of the magnetic separation mechanism with the microsieve filtration method simultaneously [34]. We used the positive selection mode to continuously separate the CTCs, combining the magnetic force with cell geometry selection. The captured cells were then used for further identification using surface-enhanced Raman scattering (SERS). The proposed device was simple in construction, enabling high throughput and high recovery, thus reducing the possibility of cross-contamination. In the magnetic filtering process, CTCs should be trapped while normal blood cells should be rinsed away. Moreover, cellular damage should be kept as low as possible for the benefit of subsequent research. The major microsieve designs fall into two categories: circular and rectangular cavities [23,35,36,37]. The other geometry varieties can be treated as the transitional status between these two scenarios. However, the influence of different pore geometries to the cell sorting efficiency and cell damage has not been demonstrated experimentally, only theoretically [38]. In this paper, two sample injection methods, which are commonly used in most recent microsieve researches, are studied to optimize the microsieve designs of constant flow rate mode and constant pressure mode. In the constant flow rate mode, a sharp rise in the fluidic pressure across the sieve can be caused by cell blockage; while in the constant pressure mode, the filtration speed can be greatly reduced as the number of cells reaching the sieve surface increases [39]. Finite element numerical simulations were performed to compare the degree of damage to trapped cells by different pore geometries (circular and rectangular) at the same inlet flow velocity. Furthermore, we compared the influence of different microsieve geometries on the MCCP, with both constant pressure and constant flow rate conditions experimentally. As described before, Hela cells with green and red fluorescent proteins (Hela-GFP and Hela-RFP) were used in the experiments to represent white blood cells (WBCs) and CTCs, respectively, for the device performance calibration.

## 2. Materials and Methods

### 2.1. System Design and Fabrication

**Figure 1:**
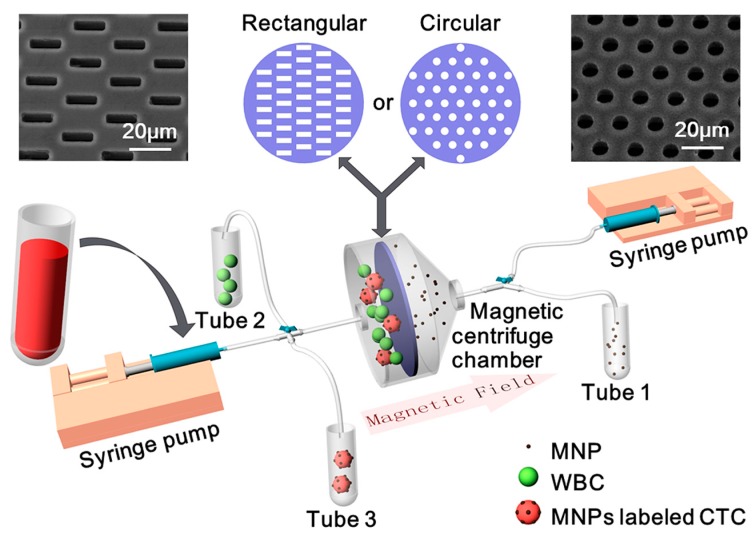
Schematic of the Magnetic Cell Centrifuge Platform (MCCP) system: a magnetic centrifuge chamber with rectangular or circular microsieves, two syringe pumps, and three collection tubes. Insert pictures show the SEM pictures of the rectangular pores (5 μm width × 15 μm length, left) microsieve and the circular pores (8 μm diameter, right) microsieve.

A schematic of our MCCP system is shown in Figure 1. The platform consists of three parts: a magnetic centrifuge chamber, two syringe pumps, and three collection tubes. Silicon-based cell microsieves with rectangular or circular pores are, respectively, sandwiched by two polydimethylsiloxane (PDMS, Sylgard 184, Dow Corning, Midland, MI, USA) layers in the magnetic centrifuge chamber to prevent leakage. This magnetic centrifuge chamber with two openings was then connected to the syringes and the collection tubes through valves and pipes, which perform not only as the inlets for supplying sample and buffer into the chambers but also as the outlets to collect the target cells and remove the waste into the tubes. By incorporating the benefits of microsieve, magnetic force, and fluidic rinse, WBCs and free MNPs in blood samples are supposed to be isolated separately, and CTCs should be enriched for subsequent investigations.

A critical condition for the identification and detection of CTCs is the ability to preserve their structural integrity and viability for cytopathological analysis. Unfortunately, the cell membranes may be damaged through contact with the edges of the microsieves. Using our previously designed 8-μm-diameter circular sieve shown in Figure 1b to separate CTCs, the abundant blood cells are easily trapped in the pores and thus cause blockage, which greatly increases the flow resistance and pressure across the surface of the sieve. Excessive pressure can lead to cell breakage and also cause damage to the sieve itself. In order to study the microsieve pore geometries’ influence on the MCCP system, we developed a new sieve with 5 μm width × 15 μm length rectangular pores, as shown in Figure 1c. Both types of sieves are perforated array structures fabricated with bulk silicon-based processes. The silicon wafer (500 μm) is first oxidized on both sides, then a 6 μm silicon nitride is deposited on the top oxide. Subsequently, the microsieve array structures are patterned and etched, stopped on the top oxide. Finally, a 5 × 5 square back-cavity was formed with the bottom native oxide acting as a hard mask. The porosity (open pore area to overall area) of the circular sieve is 11.6%, while the rectangular sieve is 9.6%.

**Figure 2:**
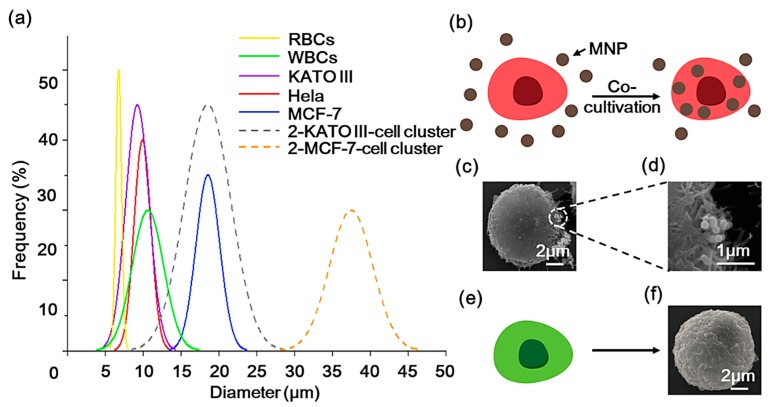
(**a**) The diameters of some typical cells are summarized (solid lines). Dashed lines display the size distribution of the two types of circulating tumor cells (CTCs) (KATO III and MCF-7) clusters with two CTCs. (**b**) Illustrations of the co-cultivation process of the red fluorescent protein (Hela-RFP) cell with the magnetic nanoparticles (MNPs), and (**c**) the SEM image of the MNPs-labeled Hela-RFP. (**d**) SEM image of the surface attached of MNPs. (**e**) The green fluorescent protein (Hela-GFP) cells used to represent white blood cells (WBCs), and (**f**) the SEM image of the Hela-GFP cell morphology.

The diameters of some typical cells are summarized in Figure 2a (solid lines) [40]. Several pieces of research have found that CTC clusters, also known as circulating tumor microemboli (CTM), have higher metastatic potential than individual CTCs. The majority of clusters consist of 2–4 individual CTCs, and may also contain cancer-associated fibroblasts, platelets, red blood cells (RBCs), and WBCs [41,42]. We assume that each cluster contains two CTCs and draw the size distribution of the two types of CTC (KATO III and MCF-7) clusters in Figure 2a with dashed lines. Due to the larger size of CTM and its potential to be magnetically labeled by antibody-based methods, the MCCP system provides a possible means for isolating CTMs. Moreover, because of the size of the overlapping region between Hela and some types of CTCs and WBCs, in this study, we used Hela-RFP cells to represent CTCs, which were combined with MNPs (200 nm in diameter, 10 mg/mL, Lmnano) by a co-cultivation process (Figure 2b). Figure 2c,d show the SEM images of the morphology of the MNPs-labeled Hela-RFP and the superparamagnetic nanoparticles attached to the cell surface that had not been endocytosed into the cells, respectively. Hela-GFP cells were used to represent WBCs (Figure 2e), and Figure 2f shows the SEM images of the morphology of the Hela-GFP. The purpose of the fluorescent staining is to determine cell types and counts subsequently.

### 2.2. Sample Preparation

Both GFP-Hela and RFP-Hela cells were cultivated in Dulbecco’s Modified Eagle Medium (DMEM, HyClone), which contained 10% fetal bovine serum (FBS, Every Green) and 1% penicillin-streptomycin in the 25 cm^2^ tissue culture flasks at 37 °C and 5% CO_2_. A total of 100 µL superparamagnetic nanoparticles solution was added into adherent RFP-Hela cells cultured for 1 day and incubated at 37 °C for at least 24 h. The sample was washed three times with phosphate buffer saline (PBS, Solarbio) to remove residual particles and metabolic waste. Adherent RFP-Hela cells were harvested using Porcine Trypsin-EDTA (HyClone) and re-suspended in the culture flask. A Magnet (0.5 T remanence) was placed under the flask and the flask was gently agitated for 1 min. RFP-Hela cells that were not magnetically labeled were then poured off to be removed. The labeled RFP-Hela cells were subsequently diluted in PBS to the desired concentration of ~10^5^ cells/mL calibrated with the hemocytometer. On the other hand, adherent GFP-Hela cells were also harvested and re-suspended at the same concentration (~10^5^ cells/mL) and mixed in proportion (1:1) to RFP-Hela cells for device testing.

### 2.3. Operation Process

**Figure 3:**
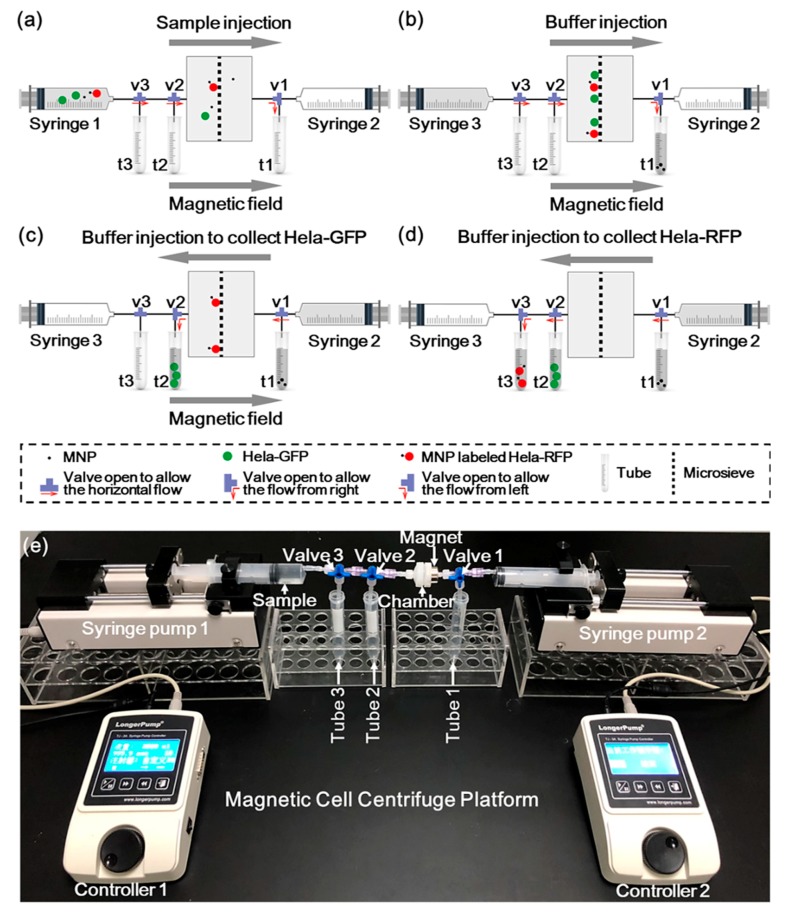
Illustration of the MCCP operation process: (**a**) The sample was injected into the chamber under a constant flow rate of 2 μL/s by syringe 1. (**b**) The buffer was injected by syringe 3 to flush the cells with that in the piping system all into the microsieve chamber. (**c**) The buffer was injected by syringe 2 to collect the unlabeled Hela-GFP with the presence of a magnet. (**d**) The buffer was injected by syringe 2 to collect the MNPs-labeled Hela-RFP. (**e**) A picture of the experimental setup with constant flow rate condition of the MCCP.

The illustration of the MCCP operation process and the experiment setup are shown in Figure 3. Figure 3a illustrates the loading of the sample. A 1 mL sample was firstly stored in syringe 1 and loaded into the chamber under a constant flow rate (2 μL/s) provided by a syringe pump (Longer TJ-3A). A permanent magnet (0.5 T remanence) was placed on the right side of the chamber to immobilize the magnetically labeled target cells on the sieve surface. Meanwhile, valves 2 and 3 were opened to allow the horizontal flow, while valve 1 was opened to allow the flow to tube 1. Sequentially, 5 mL of PBS was introduced by syringe 3, which flushed all the cells in the piping system into the microsieve chamber, as shown in Figure 3b. Valve 1 was then opened to allow the horizontal flow and valve 2 was then opened to allow the flow to tube 2, while the buffer (15 mL) flowed only from the right side supplied by syringe 2. The unlabeled cells were collected into tube 2 along with the buffer stream, as shown in Figure 3c. After removing the magnetic field, valve 2 was opened to allow the horizontal flow and valve 3 was opened to allow the flow to tube 3. The labeled cells were eventually enriched in tube 3 (Figure 3d). Our magnetic centrifuge platform enables the continuous separation of target cells from other types of substances in a sample by a single system.

Figure 3e shows the experimental setup of the MCCP in constant flow rate drive mode. The constant pressure mode can be easily implemented by replacing the syringe pumps by a constant pressure pump (WH-PMPP-112, Wenhao). Other settings were the same as the constant flow rate mode. During the subsequent experiments, cell counts were calibrated through the hemocytometer. After the separation operations, the cells in the solution in each tube were identified by a fluorescence microscope and also counted with a hemocytometer.

## 3. Results and Discussion

### 3.1. Device Modeling and Simulation

**Figure 4:**
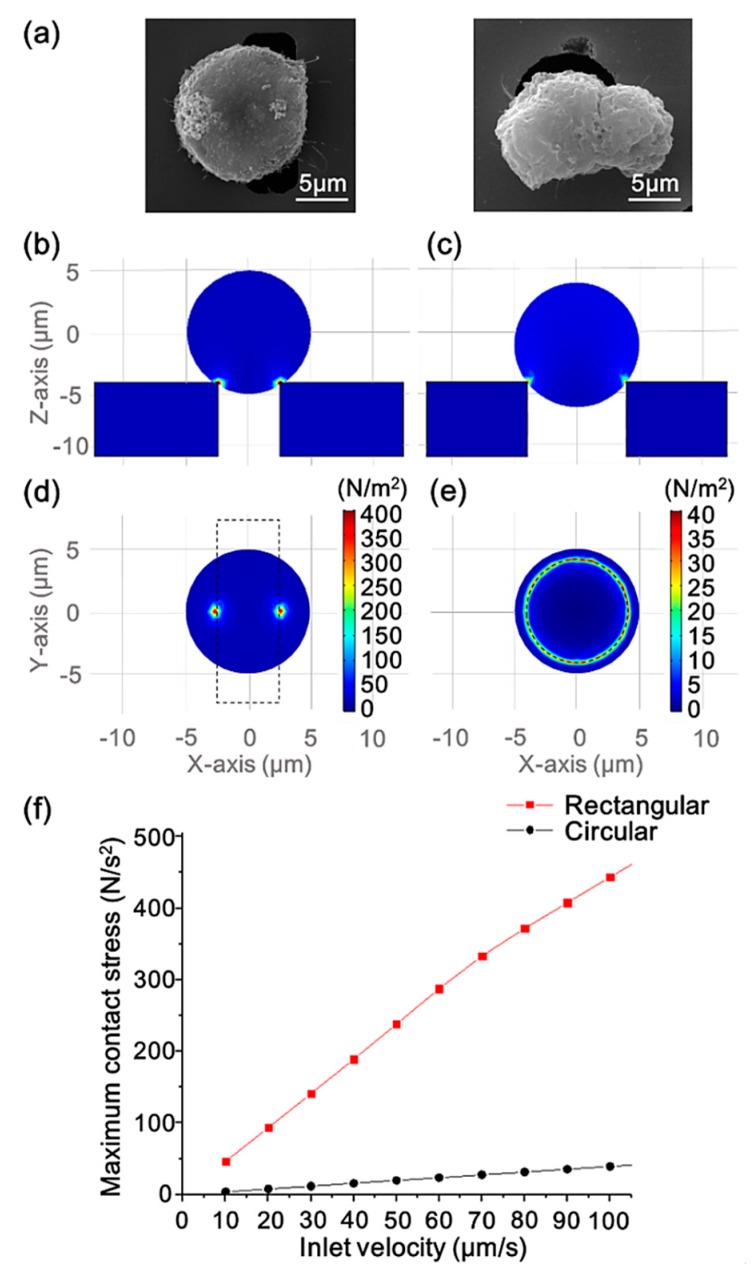
(**a**) SEM micrographs of Hela cells clogged on the rectangular (left) and circular (right) pore. (**b**) The side views of the surface contact stress of a trapped cell interacting with the rectangular pore and (**c**) with the circular pore at the inlet flow velocity of 100 μm/s. (**d**) The bottom views of the surface contact stress of a trapped cell interacting with the rectangular pore and (**e**) with the circular pore at the inlet flow velocity of 100 μm/s. (**f**) The maximum contact stress of the cell surface versus the inlet flow velocity of the two pore geometry scenarios.

Figure 4a shows SEM micrographs of a Hela cell clogged on the rectangular (left) and circular (right) pores. The comparison of the cell and the pore size can be seen in the micrographs. The diameter of the cell is longer than the short side of the rectangular pore and shorter than its long side, making the pore not completely blocked when a cell traps in it. Since the diameter of the circular pore is smaller than that of the cell, the cell will completely block the pore, making the fluid unable to pass the sieve.

FEM simulation was performed under laminar and hyperelastic conditions by COMSOL multiphysics to model the partial chamber of the rectangular pore and circular pore sieves separately to study the effect of different geometries on contact stresses when the cells were trapped in the pores. Hyperelastic theory can be used to analyze the behavior of cells [43] and Arruda–Boyce hyperelasticity coefficients were used according to the work of Boccaccio [44] in the simulations. More details concerning the FEM simulation are shown in the Supplementary Materials and Figure S1. Figure 4b–e represent flow velocity of 100 μm/s at the inlet. The side (Figure 4b,c) and bottom views (Figure 4d,e) show the surface contact stress of a trapped cell (with a diameter of 10 μm) interacting with rectangular and circular sieves, respectively. As shown in the figures, the contact area between the rectangular pore and the cell was very small, while the cell had a more balanced force distribution area when it was stuck on the circular pore. Under the action of actual fluid flow, the cells trapped on the rectangular pores were more prone to lateral displacement, and the uneven pressure would make the cells more susceptible to damage. As the inlet flow velocity gradually increased, the change in the maximum contact stress of the cells was plotted in Figure 4d. We can see that the maximum pressure generated by the contact between the cell and the rectangular pore increased sharply in the range of the flow velocity from 10 to 100 μm/s, and the maximum pressure increase in the circular pore scenario was less severe in comparison. These simulation results indicate that the sorting system with the rectangular sieve is more likely to result in cell damage.

### 3.2. Microsieve Performance and Comparison

**Figure 5:**
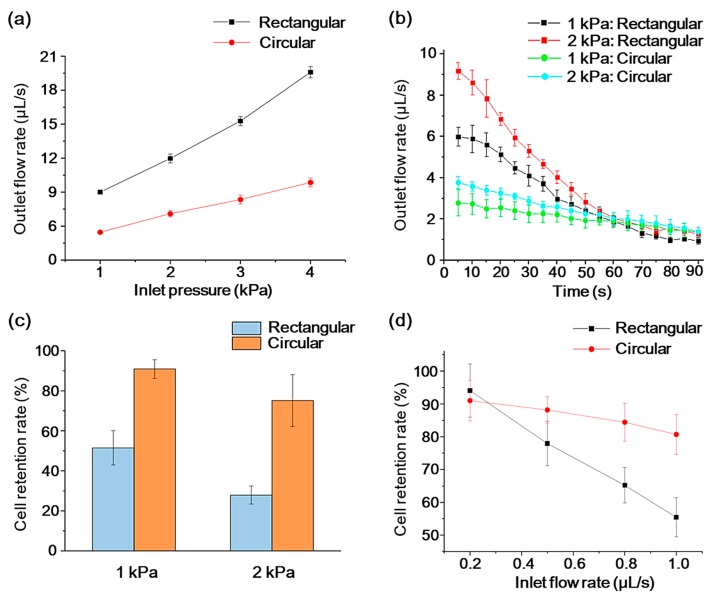
(**a**) PBS buffer flow characterization of two sieves with increasing inlet pressure. (**b**) Real-time changes in outlet flow rate with 2 × 10^5^ cells/mL buffer sample under two pressure conditions. The error bars represent the standard deviation for *n* = 3. (**c**) Cell retention rate of two sieves under two pressure conditions. (**d**) Cell retention rate of two sieves under different constant inlet flow rate. The error bars represent the standard deviation for *n* = 5.

One of the metrics for a commercial CTC separation platform is the throughput, which indicates the sample volume or number of cells that a device can handle per unit time. In this work, the flow characteristics of the two kinds of sieves were measured under different constant pressure conditions and quantified by the outlet flow rate of the chamber, which was indirectly derived from measuring the volume of liquid flowing through the chamber versus time. Figure 5a shows that when only PBS was injected into the chambers with two different sieves, the outlet flow rate increased almost linearly with an increasing inlet pressure. The rectangular sieve allowed a two- or three-times higher flow rate at the same system inlet pressure, compared with the demonstrated circular situation. This is because a smaller perimeter of a rectangular pore provides less flow resistance compared with several circular pores with the same total surface area [45].

On the other hand, the resistance of the microsieves caused by cell clogging is another critical characteristic. To characterize the clogging phenomenon of the micro sieves, buffer samples with a cell concentration of 2 × 10^5^ cells/mL were then introduced into the system, and the outlet flow rate was tested in real-time under the drive of a constant pressure pump set at 1 kPa and 2 kPa, respectively. The experimental results are shown in Figure 5b. We can see that the outlet flow rate of the rectangular sieve at the early stage of the experiment was larger than that of the circular pore under the same pressure. Meanwhile, the higher flow rate could cause faster cell accumulation on the sieve surface, so that the outlet flow rate of the rectangular sieve decreased more rapidly with time than that of the circular one. After 60 s, the flow rates tended to stabilize, thus there is not much difference between each case scenario.

Cell retention rate is another critical measure for an MCCP system. Using a constant pressure pump as the driving source, a sample containing only 4 × 10^4^ Hela cells was introduced into the chamber to assess the cell retention rate under different pressure conditions. The cell retention rate was defined as the percentage of the undamaged cells remaining in the chamber to the total number of cells. Cell morphology was observed under a microscope, and cells were manually counted through the hemocytometer. Figure 5c shows under two pressure conditions (1 kPa and 2 kPa), the Hela cells retention rate of the circular sieve outperforms that of the rectangular scenario, and the cell retention obviously decreases as drive pressure increases. These differences are partially due to the fact that the rectangular pores make it easier for the cells to deform in the longitudinal direction, causing the cells to squeeze through the sieve under hydraulic pressure [46].

The retention rate under various inlet velocity was analyzed ultimately. We used a constant flow rate syringe pump for the flow rate experiments. Figure 5d shows the change in cell retention for pores with different geometries at different constant inlet flow rates. When the inlet flow rate was slow (0.2 μL/s), the retention effect of the two sieves on cells was almost the same, at 94.1 ± 8.1% for rectangular pores, and 91.0 ± 6.2% for circular pores. At a larger flow rate, the performance of circular sieve decreased less significantly than the rectangular one. The previous simulation results predicted this smaller cell retention rate of rectangular sieves for cell damage. In addition, simulation studies showed that cells undergo periodic oscillating pressure characteristics and lead to cell damage when they pass through rectangular channels [38]. Both of these effects are the reasons why the rectangular pore may cause more cell losses.

### 3.3. Magnetically Labeled Target Cell Separation Performance and Comparison

To assess the impact of microsieves with different pore geometries on the cell sorting efficiency of the MCCP system, we quantified the system separation yield with three aspects: capture efficiency, purity, and enrichment [47]. In the following experiment, a constant flow rate (2 μL/s) was applied, considering the apparent cell retention difference and reasonable experiment duration. The Hela-GFP cells (the background cells) and the magnetically labeled Hela-RFP cells (the target cells) were mixed in the experiment sample equally for the system calibration purpose. Then, 2 × 10^4^ GFP-Hela and 2 × 10^4^ RFP-Hela cells were mixed into 1 mL buffer. The capture efficiency refers to the percentage of the cells to be sorted (S cells) that are captured from the original sample (Equation (1)). The S cells include the Hela-GFP cells in tube 2 and the Hela-RFP cells in tube 3. After the separation operations, the cells in the solution in each tube were identified by a fluorescence microscope and counted on a hemocytometer.

(1) $$\mathrm{Capture}\;\mathrm{Efficiency}\;=\frac{{\left(\mathrm{Captured}\;S\;\mathrm{cells}\right)}_{\mathrm{in}\;\mathrm{tube}\;2\;\mathrm{or}\;3}}{{\left(\mathrm{Actual}\;S\;\mathrm{cells}\right)}_{\mathrm{in}\;\mathrm{original}\;\mathrm{sample}}},$$

**Figure 6:**
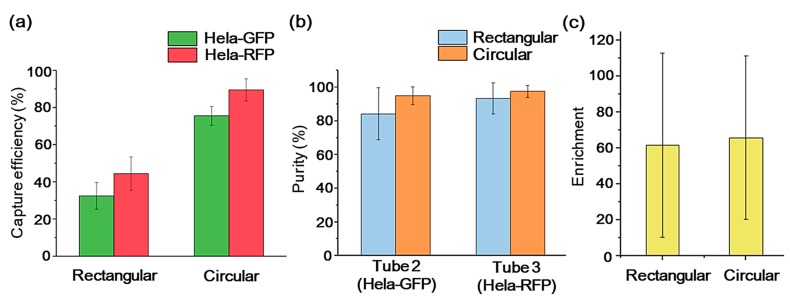
Separation yield of Hela-GFP and Hela-RFP with rectangular and circular sieves from the samples at a constant flow rate of 2 μL/s: capture efficiency (**a**), purity (**b**), and enrichment (**c**). The error bars represent the standard deviation for *n* = 5.

Figure 6a shows that the MCCP systems with rectangular and circular sieves achieved capture efficiencies of 32.4 ± 7.2% and 75.5 ± 5.1%, respectively, for Hela-GFP in tube 2, and 44.4 ± 9.0% and 89.4 ± 6.0%, respectively, for Hela-RFP in tube 3. The circular sieve outperformed its rectangular counterpart in the capture efficiency with about 133% for the Hela-GFP and about 101% for Hela-RFP. It is worth noting that the capture efficiency of the Hela-RFP was higher than that of the Hela-GFP with both kinds of microsieves. The action of the magnetic field contributes to this capture efficiency difference. During the injection of the sample under the magnetic field, the Hela-RFP cells labeled with MNPs are attracted to the silicon surface. They are less likely squeezing through the pores with buffer stream, compared to the Hela-GFP cells. Meanwhile, purity is defined as the number of the S cells captured divided by the total number of cells captured in each tube, as shown in Equation (2). Equation (3) defines the enrichment, which describes the increase in the ratio of the target cells to the background cells before and after the sample was introduced into the device. Figure 6b,c show that the MCCP system for the sorting of target cells can achieve 93.5 ± 9.3% purity and 62-fold enrichment with rectangular sieves and 97.5 ± 3.6% purity and 66-fold enrichment with rectangular sieves.

(2) $$\mathrm{Purity}\;=\frac{{\left(\mathrm{Captured}\;S\;\mathrm{cells}\right)}_{\mathrm{in}\;\mathrm{tube}\;2\;\mathrm{or}\;3}}{{\left(\mathrm{Total}\;\mathrm{captured}\;\mathrm{cells}\right)}_{\mathrm{in}\;\mathrm{tube}\;2\;\mathrm{or}\;3}},.$$

(3) $$\mathrm{Enrichment}\;=\frac{{\left(\mathrm{Captured}\;\mathrm{target}\;\mathrm{cell}/\mathrm{Captured}\;\mathrm{background}\;\mathrm{cells}\right)}_{\mathrm{in}\;\mathrm{tube}\;3}}{{\left(\mathrm{Actual}\;\mathrm{target}\;\mathrm{cells}/\mathrm{Actual}\;\mathrm{background}\;\mathrm{cells}\right)}_{\mathrm{in}\;\mathrm{original}\;\mathrm{sample}\;}},$$

**Figure 7:**
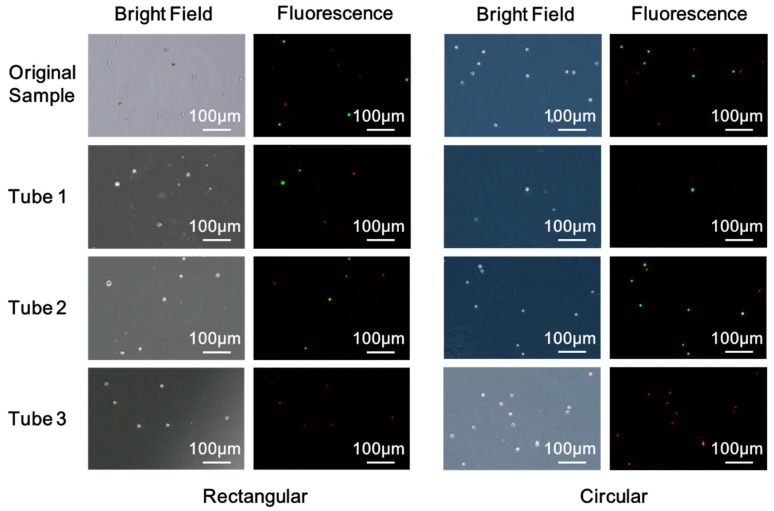
Bright field and fluorescence images of cells of the original sample and the samples collected in the tubes with rectangular and circular microsieve.

Figure 7 shows the bright field and the fluorescent images of cells under the microscope of the original sample and the samples collected in the tubes. The sample solution of each tube was centrifuged, then the supernatant was removed, leaving 0.5 mL of solution. A pipette was then used to resuspend the cells for further observation under a fluorescence microscope. Cells seen in tube 1 are due to the deformation of the cell and cell damages. The rectangular pore increases the deformation of the cell in the longitudinal direction to allow more cell penetrations. It also increases cell contact stress, leading to more cell damage and losses compared to a circular system. In addition, the action of the magnetic field contributes to fewer losses of the magnetically labeled Hela-RFP cells. Labeled cells were pinned to the sieve surface and were less likely squeezing through the pores with the buffer stream. Tube 2 is meant to collect Hela-GFP, although a small number of Hela-RFP cells could be seen in tube 2 due to the insufficient magnetic force of MNPs-labeled cells. Due to less cell damage and losses for the circular sieve in the sample injection process, it captured more and purer GFP-Hela in tube 2. Moreover, Hela-RFP cells captured in tube 3 with circular sieve outnumbered its rectangular counterpart while preserving structural integrity, as shown in Figure 7. These direct comparisons between the two pore geometries microsieve systems clearly demonstrate that circular microsieves outperform the rectangular counterparts for enriching and purifying the target cells, with a slight difference in porosity. Based on this study, the other transitional microsieve geometries aspect ratio should be optimized to avoid edge-cut damage and cell losses.

## 4. Conclusions

In this paper, we compared the geometry influence of the microsieves to the cell sorting efficiency and cell damage of the MCCP system, with constant pressure and constant flow conditions. With the circular sieve, the target cell isolation can be achieved with a purity of about 97% and enrichment of 66-fold. Notably, the MCCP system with the circular sieve outperformed its rectangular counterpart in the capture efficiency with about 101% for the target cells with a constant flow rate of up to 2 μL/s. These findings can assist the design and fabrication of the next generation of microsieves that fulfill lower cellular damage, higher volume throughput, and higher separation yield requirements. For the enrichment of the target cells, circular microsieves are recommended to be used in the MCCP due to cell retention and capture efficiency benefits.

## Supplementary Materials

The following are available online at https://www.mdpi.com/1424-8220/20/1/48/s1.
