# Supplementary material for: Magnetic Cell Centrifuge Platform Performance Study with Different Microsieve Pore Geometries
Source: Sensors (Basel). 2019 Dec 20;20(1):48. doi: 10.3390/s20010048 (PMC6983067; doi:10.3390/s20010048)
Supplement: Supplementary file 1 [file sensors-20-00048-s001.pdf]

Supplementary Materials

Details of FEM Simulations:

We combined the Laminar Flow module and the Solid Mechanics module of COMSOL to calculate contact stress between the pore and the trapped cell. A cylinder with a diameter of 25 μm and a height of 60 μm separated by sieve rectangular or circular pores with a height of 6 μm was configured to build chamber simulation geometry. The mesh resolution was selected to be 0.5 μm per lattice unit. Figure S1. shows the the geometric configuration and meshing of the simulation models of the rectangular sieve (letf) and circular sieve (left) with the trapped cells.

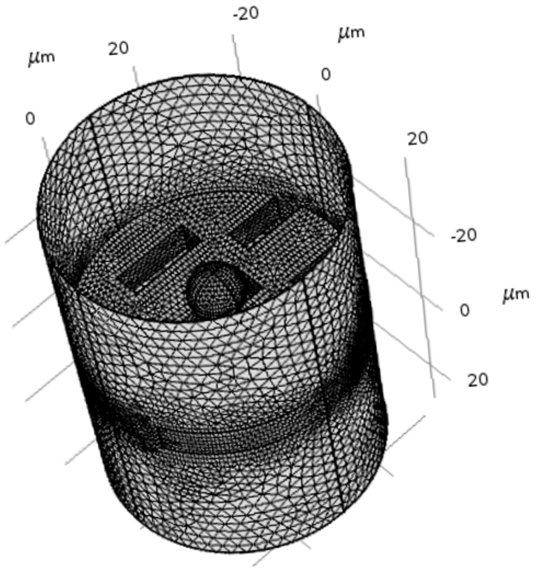

Figure S1. The the geometric configuration and meshing of the simulation models of the rectangular (letf) and circular microsieve (left) with the trapped cell.

In the Laminar Flow module, the fluid properties were set as incompressible water at 20°C (density 1000 kg/m^3 and viscosity 0.001 Pa·s). No-slip wall condition was applied on all boundaries, and normal inflow velociy was appliied on the hidden upper boundary in Figure S1 as the inlet. In the Solid Mechanics module, the cell only could move in a direction perpendicular to the plane of the sieve, fixing the displacement in other directions. Finally, the Stationary Solver was performed to compute the results.
